# “Freshman’s week”: characteristics associated with participation and experiencing adverse effects

**DOI:** 10.1186/s13011-018-0161-6

**Published:** 2018-05-29

**Authors:** E. K. Erevik, S. Pallesen, Ø. Vedaa, C. S. Andreassen, T. Torsheim

**Affiliations:** 1 Department of Psychosocial Science, University of Bergen, PO. Box. 7807, 5020 Bergen, Norway; 2 Department of Health Promotion, Norwegian Institute of Public Health, Bergen, Norway; 3 Department of Clinical Psychology, University of Bergen, Bergen, Norway

**Keywords:** Alcohol, Students, Enrolment, First year student, Adverse effects of alcohol, Personality

## Abstract

**Background:**

“Freshman’s week” (FW) is a Norwegian initiation ritual to higher education. Previous research has suggested that FW-participation is associated with better social adjustment to the student setting, as well as heavy alcohol use both during and after the event. In this study, we aimed to identify characteristics associated with participation in FW and characteristics associated with experiencing adverse effects of alcohol use during FW.

**Methods:**

Students in the city of Bergen, Norway participated in a survey during fall 2015, shortly after FW. The current sample consisted of the first-year students (*N* = 4, 401, estimated response rate: 49%). The sample’s mean age was 24 years (range: 17–73 years), 65% were females, and the majority were born in Norway (93%). Logistic regressions were conducted to identify characteristics associated with participation in FW and experiencing adverse effects.

**Results:**

A total of 64% of the first-year students reported participation in FW, and 27% of these reported experiencing at least one adverse alcohol-related effect during FW. Participation in FW was positively associated with being single (OR = 1.29), extroversion (OR = 1.18), and alcohol use (OR = 1.28), and inversely associated with age (OR = 0.70), and having children (OR = 0.36). Several characteristics (e.g., alcohol use (OR = 1.84), extroversion (OR = 0.60), symptoms of depression (OR = 1.60)) were associated with an increased risk of experiencing adverse effects of alcohol use during participation.

**Conclusion:**

The current results suggest that initiatives for increasing the participation rate in FW, reducing alcohol use during FW, and decreasing the occurrence of adverse alcohol effects during FW are warranted. Aiming to reduce the focus on alcohol use during FW, and seeking to make FW more available and enjoyable for students with other priorities, students who do not match the stereotype of the typical first-year student, and less sociable students, might both increase participation rate and prevent the occurrence of adverse alcohol effects. Future studies should aim to develop and assess interventions designed to increase participation in FW and reduce the occurrence of adverse effects related to participation.

## Background

Enrolment into higher education, like American colleges, represents an important transition in the life of emerging adults, often implying greater independency, identity reformations, and development of new social networks [1]. In Norway, students entering the first year of a new study programme of higher education (i.e., most commonly undergraduate programmes) are invited to participate in Freshman’s week (FW), which comparatively converts to American hazing or the British “Freshers’ week” in a global context. FW compromises a series of social activities and events, and typically lasts for approximately five to six days. The first-year students are typically divided into smaller groups consisting of approximately 15 first-year students. These groups are further led by about two “sponsors”, usually more senior students. The sponsors introduce games, masquerades, and parties for the new students. There are no official records of the history of FW, but the institutions for higher education in Bergen, Norway, estimate that FW has been a common initiation ritual to higher education in Bergen for at least 15 years. All larger institutions for higher education in Norway have now institutionalized FW as a part of their welcome ritual for new students. FW is mainly scheduled for evening and night time, and the public commonly associates the event with heavy drinking. Two recent studies employing a national sample of Norwegian students (different sample than the current study) have investigated demographic characteristics, social integration and alcohol use associated with FW-participation [2, 3]. Still, several questions regarding the Norwegian FW remain unanswered pertaining in particular to the questions of who is participating and who is experiencing adverse effects of FW-participation.

### Characteristics associated with participation in FW

Participation in FW has been positively associated with later social adjustment within the Norwegian setting of higher education [2]. This association might be explained by selection processes. The association between FW-participation and social adjustment might also be related to FW being the first opportunity to form friendships with co-students, hence those who do not participate might have a harder time being included in friendships groups established during FW. In this respect, students who choose not to participate in FW may find themselves in a socially disadvantageous position, and efforts to identify and arrange for these students to be included in initiation rituals such as FW may be important. Very few studies have investigated characteristics associated with participation in FW or similar initiation rituals. A recent Norwegian study showed that single and younger students were more likely to participate in FW (traits which are also related to social drinking in general), and students who abstain from alcohol were less likely to participate [2, 4]. Being born in Norway and student at a business school are examples of other characteristics that might be associated with FW-participation. Norwegian culture is traditionally regarded as a “dry” culture, where Norwegians tend to enjoy alcohol seldom while at the same time getting more intoxicated when they do drink [5, 6]. Hence, foreign-born students may shy away from FW due to past experiences with Norwegian’s heavy episodic alcohol consumption. Publicly, students at Norwegian business schools are believed to be particularly preoccupied with FW. These students have also been found to have a higher alcohol consumption than other students [7]. The emphasise made on FW and alcohol use by business students suggests that business students might be more likely to participate in FW compared to other students.

### Characteristics associated with experiencing adverse effects of alcohol use during FW

Identifying factors associated with experiencing adverse effects of alcohol use, such as impaired physical or psychological health during FW, may provide important information about students at risk. Thus, information about this could be decisive in preventing the occurrence of adverse alcohol effects among students. No previous study thus far has investigated characteristics associated with students experiencing adverse effects of alcohol use during initiation rituals to higher education. Still, characteristics associated with an increased vulnerability for experiencing adverse effects of alcohol in general, have been identified [8–15]. The most important one is likely to be the amount of alcohol consumed, as higher alcohol intake has consistently been linked to increased risk of a range of negative consequences [16, 17].

Additional factors such as gender and psychological health can also influence how individuals react to alcohol intake [8–11]. Men have consistently, and across countries, been found to report both high intake of alcohol and more adverse effects of alcohol compared to women [8]. While women have lower tolerance to alcohol than men and high alcohol intake among women may be less socially accepted. It has therefore been suggested that women experience more alcohol-related problems with the same consumption level as men [8]. Younger students may also have a higher risk of experiencing adverse effects of alcohol compared to older students, in part because alcohol could be more damaging to the developing brain [10]. Personality traits may predict both drinking and the level of adverse effects experienced. For example, one American study reported that students who scored high on neuroticism tended to report more negative consequences of alcohol use [9]. Moreover, depression and anxiety have been linked to both increased alcohol consumption and the experience of more adverse effects of alcohol use [11–15].

### Objectives

In this study, we aimed to delineate the characteristics (e.g., demographic and personality factors) associated with participation in FW. A second aim was to identify characteristics associated with experiencing adverse alcohol effects during FW (including negative effects on self-reported psychological and physical health, and feelings of being left out). We chose a survey design to address these questions. Surveys are a cost-effective way of collecting a lot of data from a number participants. We reasoned that including several variables and a high number of participants were especially important given the novelty of FW as a research topic. In addition, as the focus was on the students subjective experiences regarding the FW, we argue that the survey method is appropriate.

## Methods

### Procedures and sample

All students registered at the four largest institutions of higher education in Bergen municipality, Norway, were invited via e-mail to participate in an online survey (autumn 2015, approximately one month after the completion of FW). The institutions included one public university offering a range of subjects (University of Bergen), one public university college offering a range of subjects (named Bergen University College at the time of the data collection), one private business school (BI Norwegian Business School), and one public business school (NHH Norwegian School of Economics). Non-responders were sent up to two e-mail reminders. Those who responded to the survey took part in a lottery with two iPhone 6 s and 50 gift cards (each with a value of 500 NOK = ~ 50 EUR) as prizes.

The present study was part of a larger project investigating student health and substance use. A total of 28,553 students (from all years of study) were invited to participate in the survey, whereof 11,236 (39%) agreed to participate. The present sample included students who reported being in their freshman year in their current study programme and who answered questions regarding FW (*N* = 4401). A total of 425 students were excluded from the analyses altogether due to non-response on the items assessing FW-participation. The survey had a mandatory response design and the items assessing FW-participation were located towards the end of the survey, hence there is little missing data altogether. The included institutions have estimated that they had 5465 (the public university), 2100 (the public university college), 965 (the private business school), and 443 (the public business school) first-year students in autumn 2015, respectively. In the current sample, a total of 2136 participants were registered as students at the public university (estimated response rate 39%), 1663 were registered as students at the public university college (estimated response rate 79%), 349 at the private business school (estimated response rate 36%), and 253 at the public business school (estimated response rate 57%), respectively. The total response rate among first year students was thus calculated to 49% (4, 401/8, 973).

### Measurement

*Demographics* were measured by questions about sex (female; male), year of birth (response range: 1940–2000), duration of study at institutions for higher education (response range: 0–10+ years), being a first-year student (yes; no), place of birth (Norway; North of Europe; Other parts of Europe; Asia, Africa; South America; North America; Oceania), current religious identification (Buddhism; Hinduism; Islam; Judaism; Catholic Christianity; Orthodox Christianity; Protestant Christianity; other religion; do not identify with a religious belief), relationship status (single; in a relationship, but living alone; cohabitant; married; other), and parental status (do not have a child/children; have daily custody of a child/children; have shared custody of a child/children; have a child/children, but not custody) [18]. We discerned the student’s educational affiliation (i.e., whether they were business or college/university students) from their student e-mail addresses.

The students’ *personality* traits were assessed with the 20-item Mini International Personality Item Pool (Mini-IPIP; [19]). The Mini-IPIP is considered a reliable and valid measure of the Five-Factor Model’s personality dimensions: extroversion, agreeableness, conscientiousness, neuroticism and intellect/imagination [19]. Cronbach’s alphas for the different traits ranged from: .69–.82 (present study). The Mini-IPIP consists of statements regarding typical behaviour (e.g., being talkative, interested in others, organized, worried, imaginative), where the respondents rate the statements’ applicability of describing their behaviour. There are four statements for each of the five personality traits, each with response alternative ranging from 1 (very inaccurate) to 5 (very accurate). Thus, for each trait the composite scores range from 4 to 20.

We assessed the students’ *alcohol use* by the 10-item Alcohol Use Disorders Identification Test (AUDIT; [20, 21]) - Cronbach’s alpha: .78 (present study). AUDIT measures three dimensions of alcohol use during the past year: Consumption (i.e., frequency of drinking, typical quantity consumed, and frequency of heavy drinking), Dependency symptoms (i.e., impaired control, increased salience, and morning drinking), and Harmful alcohol use (i.e., guilt after drinking, blackouts, alcohol-related injuries, and others being concerned about the respondents’ drinking) [20, 21]. In the question regarding drinking frequency the response alternatives are: Never; once a month or less; 2–4 times a month; 2–3 times a week; 4 times a week or more. In the question regarding typical quantity consumed on a single occasion the response alternatives are: 1–2 alcohol units; 3–4 alcohol units, 5–6 alcohol units, 7–9 alcohol units; 10 or more alcohol units. In the questions regarding experiencing alcohol-related injuries and others being concerned about own drinking, the response alternatives are: No; yes, but not during the past year; yes, during the past years. For the other questions (i.e., frequency of heavy drinking, the three dependency items, guilt after drinking, and blackouts) the response alternatives are: Never; seldom; a couple of times a month; a couple of times a week; almost daily) The composite AUDIT-scores range between 0 and 40 [20, 21].

*Psychological health* were measured by the 25-item Hopkins Symptoms Checklist (HSCL-25; [22]), which measures symptoms of anxiety and depression. Cronbach’s alpha was .81 for the anxiety subscale and .89 for the depression subscale. In HSCL-25 respondents are asked about the level of different symptoms of anxiety (e.g., nervousness) and depression (e.g., loss of energy) experienced the last two weeks (response alternatives: not at all = 1; somewhat = 2; a great deal = 3; very much = 4). Total scores range between 10 and 40 for the anxiety subscale, and between 15 and 60 for the depression subscale.

The students were asked about *participation in FW*. The students were first asked if they participated in FW as new students in autumn 2015. Students who answered “yes” to this question were further asked some follow-up questions: “Did you drink alcohol during FW?” (yes; no). “How many days did you drink alcohol during FW?” (Response alternatives ranged from 0 to 14 days). “Did you participate in any alcohol-free events during FW?” (yes; no). The students were also asked if they experienced any *negative effects of alcohol during FW*. “Did you experience any of the following negative aspects of alcohol use during FW?”: a) “Impaired physical health?”, b) “Impaired psychological health?”, c) “Felt left out?”, and d) “Involved in violence/crime?” (Response alternatives: yes; no) [18].

### Analysis

All data analyses were carried out using IBM SPSS Statistics 23 (IBM SPSS Statistics, New York, USA). Missing data were deleted listwise (only relevant for the analyses in Table 4).

We started by calculating the sample’s central tendencies on the demographic, personality, alcohol use, and psychological health measurements. We also calculated the FW-participants’ central tendencies on alcohol involvement during FW and the experience of adverse effects during this week (i.e., impaired physical health, impaired psychological health, feeling left out, and involvement in violence/crime). The readers should note that the items assessing alcohol involvement during FW had some missing data. The missing data on these variables appear to be caused by technical errors related to survey administration.

A binary logistic regression was employed to investigate demographical, personality, alcohol use, and psychological characteristics of students who participated in FW compared to nonparticipating students. Participation in FW comprised the dependent variable. The independent variables were; sex (woman vs. man), age, number of years spent studying, country of birth (Norway vs. other), religion (religious vs. nonreligious), relationship status (single vs. in a relationship), parental status (have children vs. do not have children), educational institution (business institution vs. institutions offering a range of subjects), scores on the five personality traits, AUDIT-scores, and scores on depression and anxiety symptoms. The personality, alcohol use, and psychological health scores were recalculated into z-scores before being entered to the regression model.

Next, we generated three binary logistic regression models. The dependent variables were reporting impaired physical health, impaired psychological health, or feeling left out due to alcohol use during FW. The independent variables in these regression models were the same as the independent variables in the models investigating characteristics associated with participation in FW (i.e., demographics, personality, alcohol use, and psychological health variables). In addition, responses regarding alcohol involvement during FW (i.e., number of days drinking and participation in alcohol free-events) were included as independent variables. The number of days drinking during FW was transformed into z-scores before being included in the regression models. Only those reporting participation in FW were included in these analyses. In total, 289 cases (10%) were deleted from the analyses due to missing data on one or two of the included independent variables (i.e. number of days drinking and participation in alcohol free-events).

The associations between the independent and dependent variables are reported in terms of odds ratios (OR), which is considered as effect sizes. There are not clear cut-offs for how ORs should be interpreted, but ORs of 1.5, 2.5, 4.0, and 10.0 have been suggested as representing small, moderate, large, and very large effect sizes, respectively [23–25].

## Results

The sample’s characteristics are presented in Table 1. The sample’s mean age was 24 years (range: 17–73 years, *SD* = 7.1), 65% (*n* = 2, 844) were females, and the majority were born in Norway (93%, *n* = 4, 077).

**Table 1:** Characteristics of the “new” students fall 2015, *N* = 4401

	Mean (SD)/Percentage (95% CI)
Demographics	
Women	64.6% (63.2–66.0%)
Age	24.4 (7.1)
Born in Norway	92.6% (91.9–93.4%)
Religious	35.5% (34.1–37.0%)
Single	49.6% (48.1–51.1%)
Had child/children	12.1% (11.2–13.1%)
Business school affiliation	13.7% (12.7–14.7%)
Years in higher education	1.9 (2.2)
Personality^a^	
Extroversion	14.0 (3.6)
Agreeableness	16.8 (2.8)
Conscientiousness	14.6 (3.2)
Neuroticism	11.2 (3.6)
Intellect/imagination	14.6 (3.2)
Alcohol use^b^	
AUDIT-score	8.1 (4.8)
Psychological health^c^	
Depression symptoms	24.5 (7.6)
Anxiety symptoms	15.1 (4.2)

*SD* Standard deviation, *CI* Confidence interval, *AUDIT* Alcohol Use Disorders Identification Test

^a^Total scores range from 4 to 20 for each personality trait, ^b^Total scores range from 0 to 40, ^c^Total scores on the depression subscale range from 15 to 60 and total scores on the anxiety subscale range from 10 to 40

Table 2 depicts the FW-participants’ responses regarding alcohol involvement and the experience of adverse alcohol effects during FW. A total of 64% of the first-year students reported that they participated in FW, and 27% of these reported experiencing at least one adverse alcohol-related effect during FW.

**Table 2:** Alcohol use and adverse effects of alcohol use during Freshman’s week (FW), *n* = 2835 (participated in FW)

	Mean (95% CI)/Percentage (95% CI)
Alcohol use during FW	
Drank alcohol	93.6% (92.7–94.6%)^a^
Number of days drinking (among drinkers)	4.1 (2.0)^b^
Participated in alcohol-free events	45.3% (43.3–47.2%)^c^
Adverse immediate effects	
Impaired physical health	21.0% (19.5–22.5%)
Impaired psychological health	4.2% (3.4–4.9%)
Felt left out	7.2% (6.2–8.1%)
Involved in violence/crime	0.4% (0.2–0.6%)

*CI* Confidence interval, ^a^287 missing cases, ^b^289 missing cases, ^c^286 missing cases

Table 3 shows characteristics associated with participation in FW. Participation in FW was significantly (*p* < .05) and positively associated with being single, having a business school affiliation, extroversion and alcohol use, and inversely associated with age, total years spent studying and having child/ren.

**Table 3:** Characteristics associated with participating in Freshman’s week (FW), total *N* = 4401

	Participated in FW (*n* = 2835)
	(Reference category: Did not participate in FW)
	OR (95% CI)
Demographics	
Sex	
Man	1.00
Woman	0.92 (0.76–1.13)
Age	0.80 (0.78–.0.83)^***^
Total years spent studying	0.70 (0.67–0.74)^***^
Place of birth	
Born outside of Norway	1.00
Born in Norway	0.79 (0.57–1.09)
Religious identification	
Nonreligious	1.00
Religious	1.01 (0.84–1.21)
Relationship status	
In a relationship	1.00
Single	1.29 (1.08–1.53)^**^
Parental status	
Did not have child/children	1.00
Had child/children	0.36 (0.23–0.56)^***^
Educational institution	
Not business school	1.00
Business school	1.60 (1.23–2.08)^***^
Personality	
ExtroversionZ	1.18 (1.07–1.29)^**^
AgreeablenessZ	1.08 (0.99–1.19)
ConscientiousnessZ	1.01 (0.92–1.10)
NeuroticismZ	1.01 (0.90–1.13)
Intellect/imaginationZ	0.96 (0.88–1.05)
Concurrent alcohol use (AUDIT-scoreZ)	1.28 (1.16–1.41)^***^
Psychological health	
Depression symptomsZ	1.08 (0.94–1.23)
Anxiety symptomsZ	0.93 (0.83–1.05)
*Model,* df = 16 and *p* < .001	Cox & Snell R^2^ = .390; Nagelkerke R^2^ = .536

*OR* odds ratio, *CI* confidence interval, *AUDIT* Alcohol Use Disorders Identification Test, ** *p* < .01, ***p < .001, Z = z-score

Characteristics associated with reporting different adverse effects of alcohol during FW are displayed in Table 4. Total years spent studying, business school affiliation, concurrent alcohol use, depression and numbers of days drinking during FW were positively associated with reports of one or more adverse alcohol effects. Extroversion and participation in alcohol-free events during FW were inversely associated with reports of adverse alcohol effects.

**Table 4:** Characteristics associated with experiencing adverse alcohol effects during Freshman’s week (FW), total *n* = 2546 (participated in FW and had no missing data on any of the included variables)

	Impaired physical health (*n* = 595) (Reference category: Did not experience impaired physical health)	Impaired psychological health (*n* = 118) (Reference category: Did not experience impaired psychological health)	Felt left out (*n* = 204) (Reference category: Did not feel left out)
	OR (95% CI)	OR (95% CI)	OR (95% CI)
Demographics			
Sex			
Man	1.00	1.00	1.00
Woman	0.93 (0.73–1.18)	0.73 (0.45–1.17)	1.16 (0.79–1.69)
Age	1.00 (0.94–1.06)	0.98 (0.88–1.10)	0.95 (0.87–1.03)
Total years spent studying	1.04 (0.95–1.13)	1.25 (1.07–1.45)^**^	0.96 (0.84–1.10)
Place of birth			
Born outside of Norway	1.00	1.00	1.00
Born in Norway	0.99 (0.63–1.54)	0.77 (0.33–1.77)	0.93 (0.49–1.79)
Religious identification			
Nonreligious	1.00	1.00	1.00
Religious	0.83 (0.66–1.03)	0.91 (0.58–1.41)	1.00 (0.72–1.40)
Relationship status			
In a relationship	1.00	1.00	1.00
Single	0.96 (0.78–1.18)	1.00 (0.66–1.52)	0.74 (0.54–1.01)
Parental status			
Did not have child/children	1.00	1.00	1.00
Had child/children	0.71 (0.20–2.56)	1.79 (0.34–9.44)	0.26 (0.03–2.31)
Educational institution			
Not business school	1.00	1.00	1.00
Business school	1.48 (1.15–1.90)^**^	0.91 (0.54–1.51)	1.03 (0.68–1.55)
Personality			
ExtroversionZ	1.05 (0.93–1.19)	0.79 (0.64–0.99)^*^	0.60 (0.51–0.71)^***^
AgreeablenessZ	0.99 (0.88–1.11)	1.21 (0.97–1.51)	1.01 (0.86–1.19)
ConscientiousnessZ	1.12 (1.00–1.24)	1.10 (0.89–1.36)	1.06 (0.90–1.24)
NeuroticismZ	1.05 (0.92–1.20)	1.24 (0.93–1.64)	1.16 (0.94–1.44)
Intellect/imaginationZ	1.08 (0.97–1.20)	0.96 (0.78–1.18)	1.09 (0.93–1.27)
Concurrent alcohol use (AUDIT-scoreZ**)**	1.67 (1.48–1.88)^***^	1.84 (1.50–2.26)^***^	1.31 (1.10–1.56)^**^
Psychological health			
Depression symptomsZ	1.17 (1.00–1.36)^*^	1.60 (1.24–2.05)^***^	1.37 (1.13–1.68)^**^
Anxiety symptomsZ	1.05 (0.91–1.20)	1.18 (0.95–1.47)	1.19 (1.00–1.42)
FW-factors			
Number of days drinking during FWZ	1.68 (1.50–1.89)^***^	1.22 (0.98–1.51)	0.85 (0.71–1.02)
Participation in alcohol-free events			
Did not participate in alcohol-free events	1.00	1.00	1.00
Participated in alcohol-free events	0.72 (0.58–0.88)^**^	0.60 (0.38–0.93)^*^	0.62 (0.44–0.86)^**^
*Models,* df = 18 and *p* < .001 for all	Cox & Snell R^2^ = .126; Nagelkerke R^2^ = .190	Cox & Snell R^2^ = .062; Nagelkerke R^2^ = .197	Cox & Snell R^2^ = .069; Nagelkerke R^2^ = .161

*OR* odds ratio, *CI* confidence interval, *AUDIT* Alcohol Use Disorders Identification Test, * *p* < .05, ** *p* < .01, ***p < .001, Z = z-score

## Discussion

A substantial minority (i.e., 36%) of the first-year students chose not to participate in FW. Further, the emphasis on alcohol use during FW is apparent from the current results, as the vast majority of students reported drinking alcohol during the event. The mean number of days drinking was four (among drinkers). Considering that FW usually lasts for about five to six days, four days of drinking illustrates the overarching role of alcohol during this week. About one quarter of the students reported experiencing at least one adverse alcohol effect during FW.

We identified several correlates of FW-participation and reports of adverse effects related to FW-participation. Our results are largely in line with previous findings, from a range of countries, regarding characteristics associated with high alcohol use (a substantial component in FW) and vulnerability to experiencing adverse effects of alcohol use in general [4, 7, 12, 13, 16, 17, 26]. The findings regarding who participates in FW are also in accordance with two similar Norwegian studies, although the present study elaborates characteristics associated with FW-participation by including a higher number of variables than the previous studies [2, 3]. No previous study has investigated characteristics associated with experiencing adverse effects associated with alcohol use during FW or similar initiation rituals, specifically.

### Characteristics associated with participation in FW and experiencing adverse effects of alcohol use during FW

There is a wide range of plausible explanations as to why each of the identified characteristics was associated with participation in and/or experiencing adverse alcohol effects during FW. We will focus on four possible common explanations in the following sections: a) high alcohol use, b) independence from other obligations**,** c) similarity to the “typical first-year student”, and d) sociability.

#### High alcohol use

The students who reported high alcohol use in genereal were more likely to both participate in and report adverse alcohol-related effects during their participation in FW. Students who consume much alcohol may be more likely to join in on FW because they are more comfortable with the alcohol focus of FW. The association between high general alcohol use and drinking more days during FW, and reports of adverse alcohol effects may come as no surprise, as alcohol’s strain on physical and psychological health is dependent on the total amount of alcohol consumed [16, 17]. Business students’ increased likelihood of reporting impaired physical health in relation to alcohol use during FW may also relate to alcohol use in general, as these students tend to drink more and are publicly believed to be more invested in and consume more alcohol during FW, compared other students [7]. The current results suggested that participating in alcohol-free events reduced the risk of experiencing adverse effects. The students who participated in alcohol-free events might have had a lower total consumption of alcohol during FW, which may explain why they reported fewer adverse alcohol-related effects. In addition, alcohol-free activities could be more socially inclusive and less norm-breaking, and good social experiences might further act as a buffer towards adverse alcohol-related effects [27].

#### Independence from other obligations

Several of the characteristics associated with FW-participation (i.e. younger age, less experience with higher education, and being single and childless) may also be associated with having fewer social or economic responsibilities. Students with more obligations perhaps shy away from FW due to the scheduling of the event in the evening/night time (which results in FW taking up quite a lot spare time) and the heavy alcohol consumption involved (which could negatively affect obligations the next day). For instance, students with children may be less able to participate in events that take place during evening/night time due to family obligations.

#### Similarity with the “typical first-year student”

The students who chose to participate in FW were also characterised by traits (e.g. younger age) which matches the conception of the “typical first-year students” [28]. Students who do not match the stereotype of the first-year student might be hesitant to participate in FW for fear of not fitting in socially. The overrepresentation of “typical first-years students” in FW may further result in the “atypical first-year students” being ostracised. In accordance with this, students with more previous study experiences (previous study experience might be considered as at odds with the conception of the “typical first-year student”) were more likely to report negative psychological effects in relation to their FW-participation.

#### Sociability

Several of the traits related to FW-participation and experiencing adverse effects in relation to participation could also be linked to sociability. The students who participated were more likely to have traits that could be related to an increased interest in making new friendships (i.e. younger age, less experience with higher education, being single, and extroversion) [19, 29, 30]. For instance, extroversion was positively associated with FW-participation. This trait is linked with increased sociability, and enjoyment of social settings involving several individuals [19, 29], which can explain the increased participation rate among students with higher extroversion scores.

Traits inversely related to sociability (i.e. introversion and depression) were related to an increased risk of experiencing adverse effects in relation to alcohol use during FW. Introversion and depression have been associated with experiencing more distress when spending time with, at least several, others [29, 31, 32], which could explain why the more introverted and depressed students reported more negative experiences during FW. The organisation of FW, with students put into rather large social groups (approximately 15 students per group) and often given tasks demanding that they stand out, such as singing or dancing, may further increase introverts’ discomfort with the event as introverts tend to prefer smaller groups and staying in the background [19, 29]. The introverted and depressed students’ reports of discomfort in relation to FW-participation could also be related to social skills, as both introverted and depressed individuals have been found to have poorer social skills compared to extroverted and non-depressed individuals [31, 33, 34]. The introverted and depressed students’ increased likelihood of reporting negative effects in relation to alcohol use during FW may hence be explained by their struggling to be socially accepted and included. It might be particularly unfortunate that introverted students were less likely to participate in FW, and that introverted and depressed students were more likely to report negative effects in relation to their FW-participation, as these students might have benefitted the most from participating in and establishing good social relations during FW.

#### Implications

Participation in the Norwegian FW has been associated with better social adjustment within the setting of higher education [2], hence the high non-participant rate may be unfortunate. The percentage of students reporting impaired physical or psychological health, or feeling left out in relation to alcohol use during FW could be considered as worrisome. Hence, the current findings imply that measures to improve FW are warranted. Our findings suggest that initiatives to reduce alcohol use during FW, to make FW more available for students with other obligations and characteristics than the “typical first-year student”, and to make FW a better experience for students who are less sociable might be reasonable strategies for increasing the FW-participation rate and decreasing the occurrence of negative experiences. For instance, might increasing the number of day-time activities during FW, spreading the activities over more days, and arranging more alcohol-free events all be possible strategies for making FW more accessible to students with other obligations, as well as for reducing alcohol use during FW. Dividing the students into smaller groups than the present norm, and having more group-based tasks and games (as opposed to tasks requiring the individual to stand out) might be possible strategies for making less sociable students more comfortable with the event.

Our results may also have implications for other researchers investigating FW or similar events. Firstly, our results elucidate several potential common factors (e.g. age, relationship and parental status, and extroversion) in the relationship between alcohol use and FW-participation, which arguably should be included and controlled for in future studies. Furthermore, the high percentage of students reporting impaired physical health in relation to alcohol use during FW suggests that future studies should look into the seriousness and longevity of such effect in order to understand these phenomena in more depth and determine how it could be avoided. Investigating whether the adverse effects reported by the students are related to sexual assaults/unwanted sexual attention would be particularly timely. Future studies should aim to assess the effects of possible interventions designed to increase participation in initiation rituals into higher education and reduce the experience of adverse effects in relation to participation in such events.

#### Limitations

The present study had a cross-sectional design, which impedes deductions of directionality and causality. Still, several of the measured factors (e.g., demographics, personality characteristics) are likely to have existed before FW and they were measured shortly after the event which supports directional order. However, causality cannot be established by the current design as potential, unmeasured third variables may explain both the independent variables and the dependent variables. The relatively large sample size and the vast number of variables included are strengths of the current study as this decreases the risk of making type II errors and makes it less likely that the identified associations might be explained by third variables. It is important to emphasize nonetheless, that the high number of significance tests conducted also entail an elevated risk of conducting Type I errors. It should also be noted that most of the effect sizes of the observed associations were within the range of what is often considered as small or very small, although the interpretation of ORs is ambiguous. The size of the associations may limit the practical importance of our findings. We would, however, argue that identifying correlates of FW-participation and experiencing adverse effects in relation to FW-participation is important, even if these associations might be small, due the popularity of FW, the lack of research on this topic, and the potential pervasiveness of the social relations and alcohol habits established during FW [2, 3].

The measurements we used in the present study involve some limitations as well. One shortcoming is the lack of information regarding the quantities of alcohol consumed during FW, the specific amount of alcohol consumed during FW may explain the experience of some of the adverse effects. The measurements of adverse effects were also quite broad and it is hard to determine whether these were caused by or merely co-occurrences with alcohol use. Furthermore, all measurements were based on self-report, which may be influenced by social desirability and memory biases [35, 36]. Responses in web-based surveys (like the current one) may, however, be less influenced by social desirability bias than other modes of inquiry [37, 38]. Another important limitation of the self-report design is that it was the students themselves who defined themselves as first-year students. Different students may have different opinions on what constitutes a “first-year”. Some students may consider having their first year in a master programme as “first-year”, while others may not. A limitation with the current study is accordingly that the first-year students may represent a rather heterogeneous group. Moreover, some institutions mark the transition to their master programmes with a FW and the students are put in completely new social groups, while other institutions and study programmes may have a less distinct transition from bachelor to master. In this realm, we would argue that it could be regarded as reasonable to let the students decide and define their student category.

The generalisability of the current results should also be commented on. In this realm, it is important to consider the response rate. One limitation regarding this is that we do not know the exact response rate among first-year students. The estimated response rate of 49% among the first-year students is, however, within the range of what is often considered as acceptable, and the response rate is quite good compared to a similar Norwegian study and compared to the response rates obtained in online surveys internationally [18, 39]. Furthermore, we do not have any information about the students who chose not to participate in the survey, which renders the generalisability of the results somewhat unclear. The participating students had similar characteristics as those found in other studies among Norwegian students in terms of sex, age, relationship status, and alcohol use [18, 40]. This may imply that the study is generalisable to, at least, the Norwegian student population. Whether the results are generalisable to other student populations are harder to determine. FW is a local Norwegian custom. Abundance of partying and alcohol consumption seem, nevertheless, to be common denominators in the transition to higher education and student life around the world, and have been reported in countries such as the US, UK, and Denmark [1, 3, 41, 42]. In addition, alcohol habits are getting increasingly homogenous internationally, and the traditional borders between “wet” and “dry” cultures appear to be evaporating [5, 6]. From this perspective, the findings from the current study may be relevant for understanding initiation rituals to higher education in other student populations as well.

## Conclusion

Some students may feel excluded from initiation rituals and certain individuals seem to be especially vulnerable to experiencing adverse effects of drinking during initiation rituals. Students with high alcohol consumption and fewer obligations (e.g. childcare responsibilities), students with characteristics matching the stereotypical first-year student, and sociable students were more likely to participate in FW. Further, students with high alcohol consumption, students with characteristics that do not match the stereotypical first-year student, and less sociable students were more likely to report experiencing adverse alcohol effects during FW.

The current results suggest that initiatives aiming at increasing the participation rate in FW, reducing alcohol use during FW, and decreasing the occurrence of adverse alcohol effects during FW are warranted. Furthermore, the results suggest that reducing the focus on alcohol use during FW and making the event more available and enjoyable for students with several other obligations, students who differ from the “typical first-year student”, and less sociable students, might increase participation rate and prevent the occurrence of adverse alcohol effects. Future studies should aim to evaluate the effectiveness of possible interventions designed to increase participation in initiation rituals into higher education and reduce the risk of students experiencing adverse effects during such events.

## Abbreviations

AUDIT: Alcohol use disorders identification test
FW: Freshman’s week
HSCL-25: Hopkins symptoms checklist
Mini-IPIP: Mini international personality item pool
OR: Odds ratio

## References

[1] Borsari B, Murphy JG, Barnett NP (2007). Predictors of alcohol use during the first year of college: implications for prevention. Addict Behav.

[2] Myrtveit SM, Askeland KG, Knapstad M, Knudsen AK, Skogen JC (2017). The Norwegian student introductory week: who takes part, and is participation associated with better social integration and satisfaction among students?. EJHE.

[3] Myrtveit SM, Askeland KG, Knudsen AK, Knapstad M, Skogen JC (2016). Risky drinking among Norwegian students: associations with participation in the introductory week, academic performance and alcohol-related attitudes. Nord Stud Alcohol Dr.

[4] Single E, Wortley S (1993). Drinking in various settings as it relates to demographic variables and level of consumption: findings from a national survey in Canada. J Stud Alcohol.

[5] Pedersen W (2015). Bittersøtt. [Bitter sweet]. 3rd ed.

[6] Babor TF (2010). Alcohol: No ordinary commodity: Research and public policy.

[7] Erevik EK, Torsheim T, Vedaa Ø, Andreassen CS, Pallesen S. Alcohol use among business students: demographic, personality, and social correlates of increased consumption. Scand J Educ Res. 2017:1–11.

[8] Nolen-Hoeksema S (2004). Gender differences in risk factors and consequences for alcohol use and problems. Clin Psychol Rev.

[9] Baer JS. Student factors: understanding individual variation in college drinking. J Stud Alcohol. 2002;(14 Suppl):40–53. 10.15288/jsas.2002.s14.40 12022729

[10] Hermens DF, Lagopoulos J, Tobias-Webb J, De Regt T, Dore G, Juckes L, Latt N, Hickie IB (2013). Pathways to alcohol-induced brain impairment in young people: a review. Cortex.

[11] Kushner MG, Abrams K, Borchardt C (2000). The relationship between anxiety disorders and alcohol use disorders: a review of major perspectives and findings. Clin Psychol Rev.

[12] Harburg E, Davis D, Cummings KM, Gunn R (1981). Negative affect, alcohol consumption and hangover symptoms among normal drinkers in a small community. Nord Stud Alcohol Dr..

[13] Mc Kinney A, Coyle K (2006). Alcohol hangover effects on measures of affect the morning after a normal night's drinking. Alcohol Alcoholism.

[14] Grant BF, Stinson FS, Dawson DA, Chou SP, Dufour MC, Compton W, Pickering RP, Kaplan K (2004). Prevalence and co-occurrence of substance use disorders and independent mood and anxiety disorders - Results from the national epidemiologic survey on alcohol and related conditions. Arch Gen Psychiat.

[15] Dixit AR, Crum RM (2000). Prospective study of depression and the risk of heavy alcohol use in women. A J Psychiat.

[16] Rehm J, Room R, Graham K, Monteiro M, Gmel G, Sempos CT (2003). The relationship of average volume of alcohol consumption and patterns of drinking to burden of disease: an overview. Addiction.

[17] Corrao G, Bagnardi V, Zambon A, La Vecchia C (2004). A meta-analysis of alcohol consumption and the risk of 15 diseases. Prev Med.

[18] Nedregård T, Olsen R. Studentenes helse- og trivselsundersøkelse 2014. Students' Health and Wellbeing Survey. 2014:2014. http://www.studentvelferd.no/dokumenter/2014/09/SHoT-2014_Rapport_.pdf

[19] Donnellan MB, Oswald FL, Baird BM, Lucas RE (2006). The mini-IPIP scales: tiny-yet-effective measures of the big five factors of personality. Psychol Assessmeent.

[20] Babor TF, Higgins-Biddle JC, Saunders JB, Monteiro MG. The alcohol use disorders identification test: guidelines for use in primary care 2001. whqlibdoc.who.int/hq/2001/WHO_MSD_MSB_01.6a.pdf.

[21] Bohn MJ, Babor TF, Kranzler HR (1995). The alcohol use disorders identification test (AUDIT): validation of a screening instrument for use in medical settings. J Stud Alcohol.

[22] Derogatis LR, Lipman RS, Rickels K, Uhlenhuth EH, Covi L (1974). Hopkins symptom checklist (HSCL): self-report symptom inventory. Syst Res Behav Sci.

[23] Chen H, Cohen P, Chen S (2010). How big is a big odds ratio? Interpreting the magnitudes of odds ratios in epidemiological studies. Commun Stat-Simul C.

[24] Durlak JA (2009). How to select, calculate, and interpret effect sizes. J Pediatr Psychol.

[25] Rosenthal JA (1996). Qualitative descriptors of strength of association and effect size. J Soc Serv Res.

[26] Erevik EK, Pallesen S, Vedaa Ø, Andreassen CS, Torsheim T (2017). Alcohol use among Norwegian students: demographics, personality and psychological health correlates of drinking patterns. Nord Stud Alcohol Dr..

[27] Steele CM, Josephs RA (1990). Alcohol myopia: its prized and dangerous effects. Am Psychol.

[28] Justice EM, Dornan TM (2001). Metacognitive differences between traditional-age and nontraditional-age college students. Adult Ed Q.

[29] McCrae RR, John OP (1992). An introduction to the 5-factor model and its applications. J Pers.

[30] Pittman LD, Richmond A (2008). University belonging, friendship quality, and psychological adjustment during the transition to college. J Exp Educ.

[31] Segrin C (2000). Social skills deficits associated with depression. Clin Psychol Rev.

[32] Eysenck H (2006). The biological basis of personality. 1rd.

[33] Anderson C, John OP, Keltner D, Kring AM (2001). Who attains social status? Effects of personality and physical attractiveness in social groups. J Pers Soc Psychol.

[34] Argyle M, Lu L (1990). Happiness and social skills. Pers Indiv Differ.

[35] Tourangeau R, Yan T (2007). Sensitive questions in surveys. Psychol Bull.

[36] Raphael K (1987). Recall bias - a proposal for assesment and control. Int J Epidemiol.

[37] Bowling A (2005). Mode of questionnaire administration can have serious effects on data quality. J Public Health.

[38] Gnambs T, Kaspar K (2015). Disclosure of sensitive behaviors across self-administered survey modes: a meta-analysis. Behav Res Methods.

[39] Sheehan KB (2001). E-Mail survey response rates: a review. J Comput-Mediat Comm.

[40] Statistisk sentralbyrå [Statistics Norway]. Studenter i høyere utdanning [Students in High Educ]. 2017. https://www.ssb.no/utdanning/statistikker/utuvh.

[41] Groves M, Griggs G, Leflay K (2012). Hazing and initiation ceremonies in university sport: setting the scene for further research in the United Kingdom. Sport Soc.

[42] Larsen EL, Smorawski GA, Kragbak KL, Stock C (2016). Students’ drinking behavior and perceptions towards introducing alcohol policies on university campus in Denmark: a focus group study. Subst Abuse Treat Pr.

